# Isolation and characterization of Japanese encephalitis virus genotype I from pig provides evidence for the presence of the virus in nasal secretion and co-circulation of JEV genotypes in Assam, India

**DOI:** 10.3389/fcimb.2026.1787655

**Published:** 2026-03-03

**Authors:** Harlipura Basavarajappa Chethan Kumar, Seema Rani Pegu, Bommanahalli Ramakrishna Asha, Narayanaswamy Pradeep, Rai Vishal, Jagadish Basayya Hiremath, Gundallahalli Bayyappa Manjunatha Reddy, Sharanagouda Siddanagouda Patil, Vivek Kumar Gupta

**Affiliations:** 1 ICAR-National Institute of Veterinary Epidemiology and Disease Informatics, Bengaluru, Karnataka, India; 2 ICAR-National Research Centre on Pig, Guwahati, Assam, India

**Keywords:** Assam, genotype I, India, Japanese encephalitis virus, nasal swab, pig, virus isolation

## Abstract

**Background:**

Japanese encephalitis (JE) is a leading cause of viral encephalitis, among children, in many Asian countries despite the availability of effective vaccines. There are five JE virus genotypes (GI through GV), with GIII being the most prevalent. However, in the past three decades GI has emerged as the dominant genotype across several Asian countries, while the reappearance of GV is a concern due to the reduced cross-neutralization offered by existing GIII-based vaccines. Although both GI and GIII have been reported to co-circulate in India, all previous JEV isolations from pigs have been of the GIII.

**Objective:**

The objective of the study was to elucidate the JEV genotype diversity among pigs in Assam through molecular and virological investigation.

**Methods:**

We collected blood, serum and nasal swab samples from apparently healthy pigs as a part of routine disease surveillance in pigs of Kamrup (Rural) district, Assam, India. The samples were processed using standard molecular biology (qRT-PCR, Gene Sequencing, Phylogenetic analysis) and virological techniques (Virus isolation, immunofluorescence, plaque assay) for JE virus detection, isolation and characterization.

**Results:**

In this study, we report the first isolation and characterization of a JEV GI from a nasal swab of a naturally infected pig from Assam, India and the isolate was designated as JEV/Pig/Assam/NIVEDI-1/2025 (GI). The identity of the JEV isolate was confirmed by RT-qPCR, phylogeny based on *5′UTR–prM* region, full-length envelope protein gene, and immunofluorescence assay. The isolate reached a peak titer of 10^6.5^ TCID_50_/mL at 72 h post-infection in Porcine stable kidney cells and produced smaller plaques (1.88 ± 0.56 mm) than the reference GIII strain (2.68 ± 0.48 mm) (*p* < 0.01).

**Conclusions:**

The findings underscore that JEV GI is circulating in Assam and there is need for strengthened JEV surveillance in swine to monitor genotype shifts, understand viral evolution, and generate field isolates critical for vaccine evaluation and preparedness against emerging JEV genotypes. The study also demonstrates the feasibility of using nasal swabs for virus detection and isolation thereby providing evidence for the presence of JEV in nasal secretion of naturally infected pigs.

## 1 Introduction

Japanese encephalitis virus (JEV), the causative agent of Japanese encephalitis (JE), remains a leading cause of acute encephalitis syndrome (AES) in India (Tandale et al., 2023). Majority of the human JE cases are reported during monsoon and post-monsoon season although year-round circulation has been reported in tropical endemic regions of Asia. The disease predominantly affects children under 15 years of age; however, individuals of any age can get affected. Majority of the people infected with JEV remain asymptomatic. However, nearly 50% of the patients recovered from JE suffer from permanent neurological deficits. Globally, JEV has been estimated to cause 56,847 cases (95% CI: 18,003–184,525) and 20,642 deaths (95% CI: 2,252–77,204) in 2019 (Moore, 2021). In India, approximately 402.1 million people (range: 220.6–691.4 million) are considered at risk of JE infection (Moore, 2021). The virus is maintained in an enzootic cycle involving ardeid birds and mosquitoes, which act as reservoir and vector hosts, respectively (Sewgobind et al., 2022). Safe and effective vaccines for human use are available and are being administered in several JE-endemic countries, including India (Vannice et al., 2021).

JEV belongs to the family Flaviviridae and the genus Orthoflavivirus. It is an enveloped virus, measuring approximately 40–50 nm in diameter, with a single-stranded, positive-sense RNA genome of about 11 kb.

Based on nucleotide sequence analysis of the pre-membrane-capsid region and the full-length envelope gene, JEV isolates are classified into five (GI–GV) genotypes (Chen et al., 1992; Solomon et al., 2003). Most reported JEV isolates belong to genotypes I (GI) and III (GIII). Currently available GIII-based human and animal vaccines provide cross-protection against heterologous genotypes, though the protective efficacy is highest against homologous genotypes when compared to heterologous genotypes (Fan et al., 2022). Over recent decades, JEV GI has emerged as the dominant genotype across Asia, gradually replacing GIII in several countries (Yun et al., 2010; Schuh et al., 2014). Genotype V (GV), initially identified in 1955, re-emerged nearly six decades later in Tibet (2009) and South Korea (2010) (Takhampunya et al., 2011; Li et al., 2014; Gao et al., 2019). Such genotype shifts are likely to be influenced by viral evolution, ecological changes, vector dynamics, and migratory bird movements, underscoring the importance of continuous active genotypic surveillance.

In India, both GI and GIII genotypes have been reported in humans, pigs, and mosquitoes (Fulmali et al., 2011; Datey et al., 2020; Mote et al., 2023). However, most previous reports on genotype circulation have been based on human and mosquito samples, with limited systematic information on the genotypes currently circulating in the pig population, the key amplifying host for sustaining JEV transmission. Although isolation of GIII from pigs has been documented previously, there are no published reports of JEV GI isolation from pigs in the country. This knowledge gap limits understanding of the evolving viral ecology and genotype shift dynamics in animal reservoirs, which are critical for effective surveillance, vaccination, and control strategies. This study reports, for the first time, the isolation and characterization of JEV GI from a naturally infected pig in Assam, India. This provides crucial evidence of ongoing viral evolution at the livestock–vector–human interface and emphasizes the need for continued integrated surveillance to mitigate future transmission risks.

## 2 Materials and methods

### 2.1 Ethics approval

This study was approved by the Committee for Prevention and Supervision of Experiments on Animals (CPCSEA) with the approval number V-11011(13)/7/2025-CPCSEA-DADF in 2025.

The samples were collected by the trained veterinarian following the national animal ethical guidelines of the Government of India. Informed verbal consent was obtained from pig farmers before the sample collection.

### 2.2 Study site and sample collection

**Figure 1:**
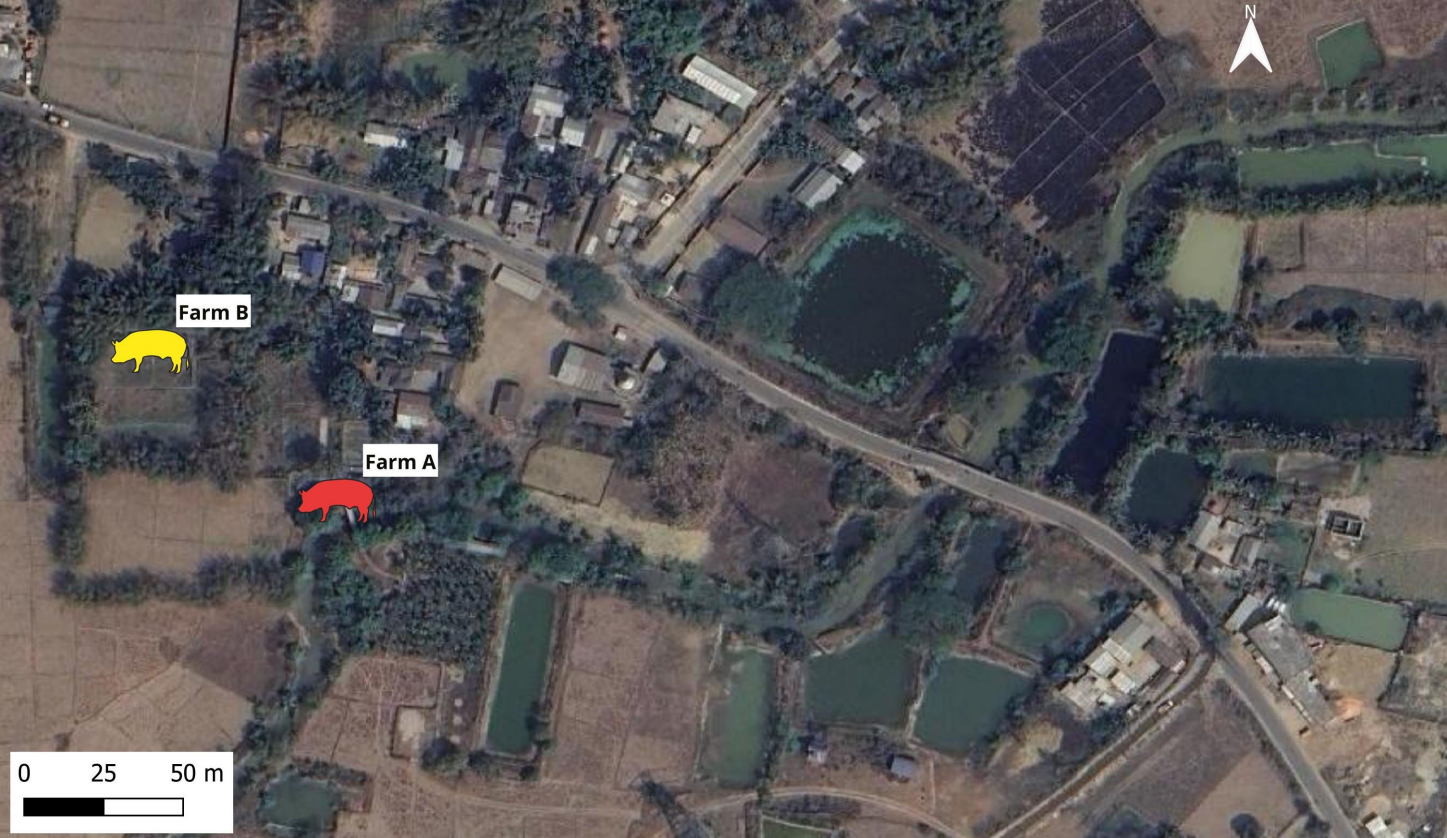
Location and ecological features around the JEV RT-PCR positive pig farms.

During May 2025, following notification by the local public health department regarding suspected JE activity in Goshaihaat village, Kamrup (Rural) district, Assam, India, ICAR-National Research Centre on Pig (ICAR-NRC on Pig), Guwahati and ICAR-National Institute of Epidemiology and Disease Informatics (ICAR-NIVEDI), Bengaluru conducted JEV surveillance among pigs in the village. The villagers maintained Large White Yorkshire and Duroc crossbred pigs and all the animals were clinically healthy at the time of sampling. In the first-round, blood samples from pigs (n=34) of 17 households were screened for JEV infection between 21–23 May 2025. Of the samples tested, pigs from two farms (hereafter mentioned as Farm A and Farm B were found positive for JEV by RT-PCR (**Table 1**, **Figure 1**). Based on these results second round of samples (n=8) were collected for testing from those two JEV positive farms on 29 May 2025. Whole blood, serum, and nasal swab samples were collected aseptically from pigs following standard biosafety protocols. The farm- and animal-level details of JEV RT-PCR positive farms are provided in **Table 2**.

**Table 1:** Sampling details of the study village.

Date of sample collection	Number of pigs sampled	Number positive by RT-PCR (%)	Serological positive (%)
2025 May 21-23	34	7 (20%)	Not done
2025 May 29	8	2 (25%)	0(0%)
2025 Jun 9-11	30	Not done	11 (37%)

**Table 2:** Farm and animal details of JEV RT-PCR positive pig farms sampled on 2025 May 29.

Farm ID and geographic coordinates	Herd size	Pig no.	Age (months)	Clinical samples collected		
				Nasal swab	Whole blood	Serum
Farm A (Lat 26.063363, Long 91.543486)	18	1	4-5	✓	✓ (+)	✓
		2	4-5	✓	✓	✓
		3	4-5	✓	✓	✓
		4	4-5	✓ (+)	✓ (+)	✓
		5	4-5	✓	✓	Sample not available
		6	4-5	✓	✓	Sample not available
Farm B (Lat 26.063833, Long 91.542899)	11	7	6-7	✓	✓	Sample not available
		8	6-7	✓	✓	Sample not available

✓= Indicates sample available for screening, (+) = Indicates RT-PCR positive specimen.

In the third round of sample collection, 30 pig serum samples were collected from the same village during 9–11 June 2025 including the Farm A and B. The samples collected on 21–23 May 2025 and June 2025 were screened at ICAR-NRC on Pig and those collected on 29 May 2025 were screened at ICAR-NIVEDI.

### 2.3 Virus and cell lines

Japanese encephalitis virus strain JEV/eq/India/H225/2009 (H225) isolated from horse and porcine stable kidney (PS) cells were kind gift from Virology Laboratory, ICAR-National Research Centre on Equines, Hisar. JEV SA14-14–2 vaccine was obtained from Department of Health Services, Government of Karnataka. Vero and PS cell lines were maintained in Eagle’s minimal essential medium (MEM; Gibco, USA) supplemented with 10% fetal bovine serum (FBS), 100 units/mL penicillin, 100 μg/mL streptomycin, and 0.25 μg/mL amphotericin-B. The cultures were incubated at 37 °C in a humidified atmosphere with 5% CO_2_ and routinely monitored for cell viability and contamination.

### 2.4 RNA extraction

Total RNA was extracted from whole blood, nasal swabs and cell culture supernatant using NucleoSpin RNA Blood Mini kit (Macherey-Nagel, Germany), and QIAamp Viral RNA Mini Kit (QIAGEN, Hilden, Germany) following manufacturer’s protocols.

### 2.5 Reverse transcription polymerase chain reaction

The cDNA was synthesized from 10 µl total RNA using random hexamers and RevertAid™ M-MuLV Reverse Transcriptase (Thermo Scientific, USA), as per the manufacturer’s instructions. Detection of JEV was carried out using both conventional and quantitative reverse transcription PCR (RT-PCR) assays. A two-step conventional RT-PCR targeting the *NS3* gene, *5′ UTR–prM* region and the Envelope gene regions, and a one-step RT-qPCR targeting the *NS2A* gene were performed as described previously (Tanaka, 1993; Yeh et al., 2010; Jeong et al., 2011; Bharucha et al., 2018). The one-step RT-qPCR was performed in a 20 µL reaction volume using the AgPath-ID One-Step RT-PCR Kit (Applied Biosystems, USA). Each reaction contained 600 nM of forward and reverse primers, 300 nM of probe, and 6.2 µL of extracted RNA template. The amplification protocol consisted of reverse transcription followed by 40 amplification cycles.

RNA extracted from cell culture supernatant of PS cell line infected with JEV SA 14-14–2 strain was used as positive control, while nuclease-free water as negative control in each RT-PCR run. Amplicons obtained from the conventional RT-PCR were visualized by agarose gel electrophoresis, purified using standard gel extraction procedures, and subjected to Sanger sequencing for confirmation of JEV identity.

### 2.6 Isolation and identification of JEV

Samples that tested positive for JEV by conventional RT-PCR were subjected to virus isolation in cell culture. Briefly, 500 µL of whole blood or nasal swab homogenate, filtered through a 0.22 µm syringe filter, was inoculated onto a sub-confluent monolayer of Vero cells in a T-25 flask. Virus adsorption was allowed for 1 h at 37 °C with intermittent gentle rocking. After adsorption, the inoculum was removed, and the cell monolayer was washed with phosphate-buffered saline (PBS, pH 7.4) to remove unbound virus. Subsequently, 5 mL of Eagle’s Minimum Essential Medium (EMEM) supplemented with 2% fetal bovine serum (FBS) was added, and cultures were incubated at 37 °C in a 5% CO_2_ atmosphere. Infected flasks were monitored daily for cytopathic effects (CPE), characterized by cell rounding, detachment, and aggregation. Each sample underwent up to four blind passages before being declared negative for virus isolation. When CPE involved approximately 70% of the cell monolayer, the culture flask was frozen at −80 °C and subjected to three freeze–thaw cycles to release intracellular virus. The culture supernatant was clarified by centrifugation at 200 × g for 10 minutes at 4 °C, aliquoted, and stored at −80 °C for further analysis. The presence of JEV in the culture supernatant was confirmed by both conventional RT-PCR, RT-qPCR, gene sequencing, and indirect immunofluorescence assay (IFA).

### 2.7 Immunofluorescence assay

The indirect immunofluorescence assay (IFA) was performed using PS cells infected with the fifth passage of the JEV isolate to confirm viral antigen expression. Briefly, a confluent monolayer of PS cells in a 25 cm² culture flask was infected with the JEV isolate and incubated at 37 °C for 48 h. Following incubation, the cell monolayer was fixed with 2 mL of ice-cold 80% acetone for 10 min at 4 °C and air-dried. The fixed monolayer was rinsed with 3 mL of phosphate-buffered saline (PBS, pH 7.4) for 5 min at room temperature.

Blocking was carried out using 2 mL of blocking buffer (PBS containing 4% bovine serum albumin [BSA] and 0.05% Tween-20) for 1 h at room temperature, followed by permeabilization with 2 mL of 0.2% Triton X-100 in PBS for 10 min. The cells were then washed three times with 5 mL of washing buffer (PBS containing 2% BSA and 0.01% Tween-20). The JEV NS4B polyclonal antibody (PA5-32211, Invitrogen, USA), diluted 1:2000 in blocking buffer was added (3 mL) and incubated for 1 h at room temperature. After washing three times with washing buffer, the cells were incubated with goat anti-rabbit IgG (H+L) cross-adsorbed secondary antibody conjugated with Alexa Fluor™ 488 (A-11008, Invitrogen, USA) diluted 1:1000 in blocking buffer (3 mL) for 1 h at room temperature in the dark. The monolayer was washed thrice with washing buffer, and 1 mL of mounting medium (90% glycerol in PBS, 0.1 M phosphate buffer) was added. Fluorescence was visualized under a fluorescence microscope. A mock-infected flask served as a negative control, and a flask infected with the JEV SA 14-14–2 vaccine strain served as a positive control.

### 2.8 *In-vitro* growth kinetics study

The *in-vitro* growth kinetics of the JEV isolate (passage 5) was assessed to determine its replication pattern in PS cells. Multiple 25 cm² flasks containing sub-confluent PS cell monolayers were infected with the JEV isolate at a multiplicity of infection (MOI) of 0.01. The cells were maintained at 37 °C in a humidified incubator with 5% CO_2_. At 24 h intervals post-infection (0, 24, 48, 72, 96, and 120 h), one flask was frozen at −80 °C. Each flask underwent three freeze–thaw cycles to release intracellular virions, and the culture supernatant was clarified by centrifugation at 200 × g for 10 minutes at 4 °C. The clarified supernatants were aliquoted and stored at −80 °C till further processing. Viral RNA was extracted from supernatant at each time-point sample and subjected to RT-qPCR. Additionally, the clarified supernatants were titrated in 96-well tissue culture plates twice using the Reed–Muench method (Reed and Muench, 1938), and the viral titers were expressed as 50% tissue culture infectious dose per milliliter (TCID_50_/mL).

### 2.9 Phenotypic characterization of viral plaques

The plaque morphology and size of the isolated JEV were compared with those of the reference strain JEV/eq/India/H225/2009 (H225). For the plaque assay, each well of a 6 well plate was seeded with 4 × 10^5^ PS cells and incubated to achieve approximately 90% confluency. The monolayers were infected with 100 µL of an appropriate dilution of either the field JEV isolate (passage 6) or the reference H225 strain (passage 14). After 1 h of virus adsorption at 37 °C, the inoculum was removed, and the wells were washed with PBS. The cell monolayers were then overlaid with a mixture of 2× MEM containing 2% FBS, antibiotics, and 0.6% agarose (yielding a final agarose concentration of 0.3%). The plates were incubated at 37 °C with 5% CO_2_ for 96 h. At the end of incubation, the cells were fixed with 10% formal saline for 1 h and stained with 1% crystal violet for 15 min. After rinsing with water and airdrying, plaque morphology and size were recorded. The diameters of 20 randomly selected plaques were measured independently by three observers, and the mean plaque size was calculated. The plaque assay was repeated thrice, and the values were expressed as Mean ± SD.

### 2.10 Phylogenetic analysis

The template cDNA was amplified using the primers targeting the nucleotide sequence of the 5′ *UTR–prM* region (505 bp) (Jeong et al., 2011) (fourth cell passage) and full-length envelope gene (1500 bp) (sixth cell passage) (Yun et al., 2010). The PCR amplicons were purified using gel purification and sanger sequencing was performed from a commercial nucleotide sequencing service provider. The sequences were aligned with corresponding regions of JEV reference sequences representing different genotypes (GI–GV) from India and other countries retrieved from the GenBank database. Nucleotide and amino acid similarity matrix was constructed in Bioedit software. Multiple sequence alignment was performed using the MUSCLE algorithm implemented in MEGA 11 software (11.0.13) (Tamura et al., 2021). The phylogenetic tree was constructed using the Maximum Likelihood (ML) method with 1000 bootstrap replicates. The Kimura 2 parameter substitution model with a discrete Gamma distribution (+G) (for 5′ *UTR–prM* region) and Tamura-Nei model with discrete Gamma distributed with invariant sites (G+I) (for Envelope gene) was applied to account for rate variation among sites (Kimura, 1980). The tree was rooted using the Murray Valley encephalitis virus (GenBank accession number NC_000943) as the outgroup. The robustness of clades was assessed by bootstrap support values, and genotypic clustering was interpreted based on established JEV genotype references.

### 2.11 Serological analysis

The serum samples collected during 21–23 May 2025, were subjected to enzyme linked immune-sorbent assay at ICAR-NRC on Pig, Guwahati and those collected on 29 May 2025 were subjected to microneutralization test at ICAR-NIVEDI, Bengaluru. The microneutralization test (MNT) was performed on pig serum samples to detect neutralizing antibodies against JEV, following the protocol described previously (Gulati et al., 2011). Serum samples were heat inactivated at 56 °C for 30 minutes and serially diluted in 2% MEM in 50 µL volumes, starting with a fivefold dilution followed by twofold serial dilutions to yield a dilution series from 1:5 to 1:320. To each diluted serum sample, 50 µL of JEV suspension containing approximately 300 TCID_50_ was added, and the serum–virus mixtures were incubated at 37 °C for 1 h. The mixtures were then added to preformed PS cell monolayers in 96well plates and incubated at 37 °C with 5% CO_2_ for 4 days. Each assay included cell control, serum control, virus back titration control, positive control serum, and negative control serum. The reciprocal of the highest serum dilution that completely inhibited the formation of cytopathic effect (CPE) was recorded as the MNT titer. Samples with titers ≥10 were considered positive for JEV specific neutralizing antibodies.

## 3 Results

### 3.1 Detection of JEV RNA

Of the 34 pigs tested between 21–23 May 2025, seven pigs belonging to two farms (Farm A and Farm B) were found positive by partial envelope gene-based RT-PCR. Based on the preliminary results, the follow-up samples were collected from farm A and B on 29 May 2025 and screened. Of these, blood and nasal swab samples from two pigs (Pig no. 1 and 4) of Farm A were found positive for JEV RNA by *NS3* gene-based RT-PCR, while samples from Farm B were negative for JEV (**Table 2**). The identity of the PCR amplicons was confirmed by gene sequencing.

### 3.2 JEV isolation and identification

**Figure 2:**
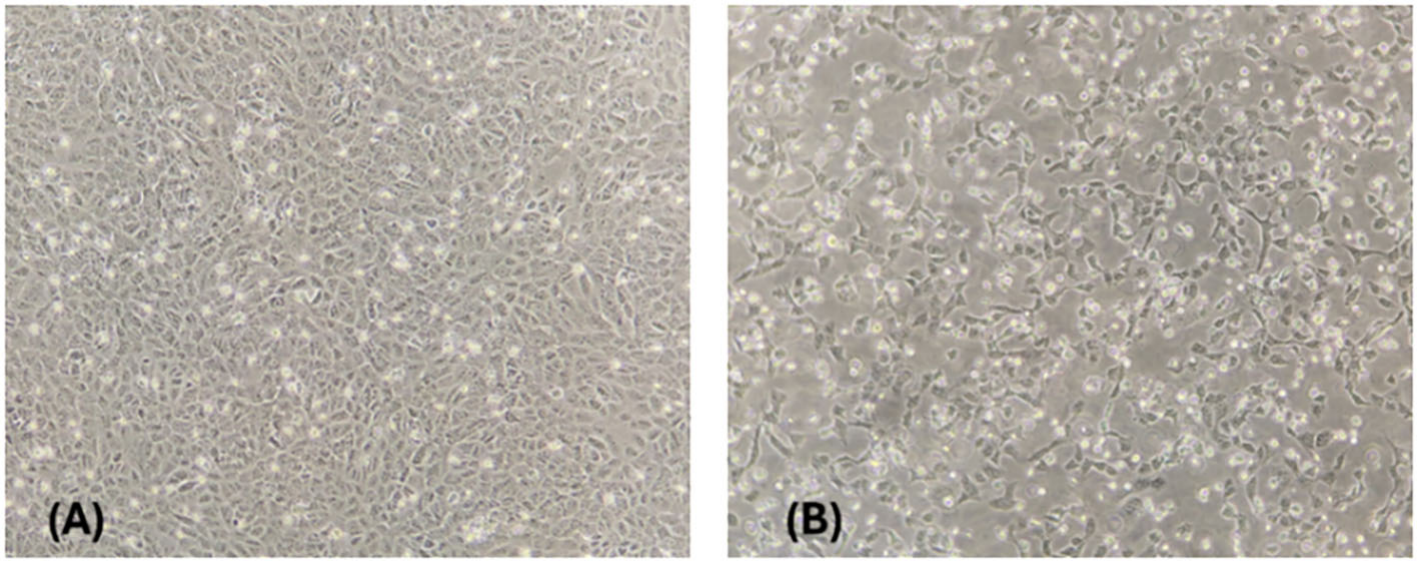
Isolation of Japanese encephalitis virus in vero cells. **(A)** Mock infected (10x) **(B)** CPE induced by JEV isolated from nasal swab of pig in Passage 2 (10x).

The RT-PCR–positive blood (n=2) and nasal swab sample (n=1) collected on 29 May 2025 from pig no. 1 and 4 of farm A were subjected to virus isolation in Vero cell culture. The Vero cells inoculated with nasal swab fluid from Pig No. 4 exhibited characteristic CPE including cell rounding and detachment, visible at the second passage (**Figure 2**). The isolate was successfully maintained through six passages and the strain was designated as JEV/Pig/Assam/NIVEDI-1/2025 (GI). However, two RT-PCR–positive blood samples did not yield viable virus even after four serial passages.

**Figure 3:**
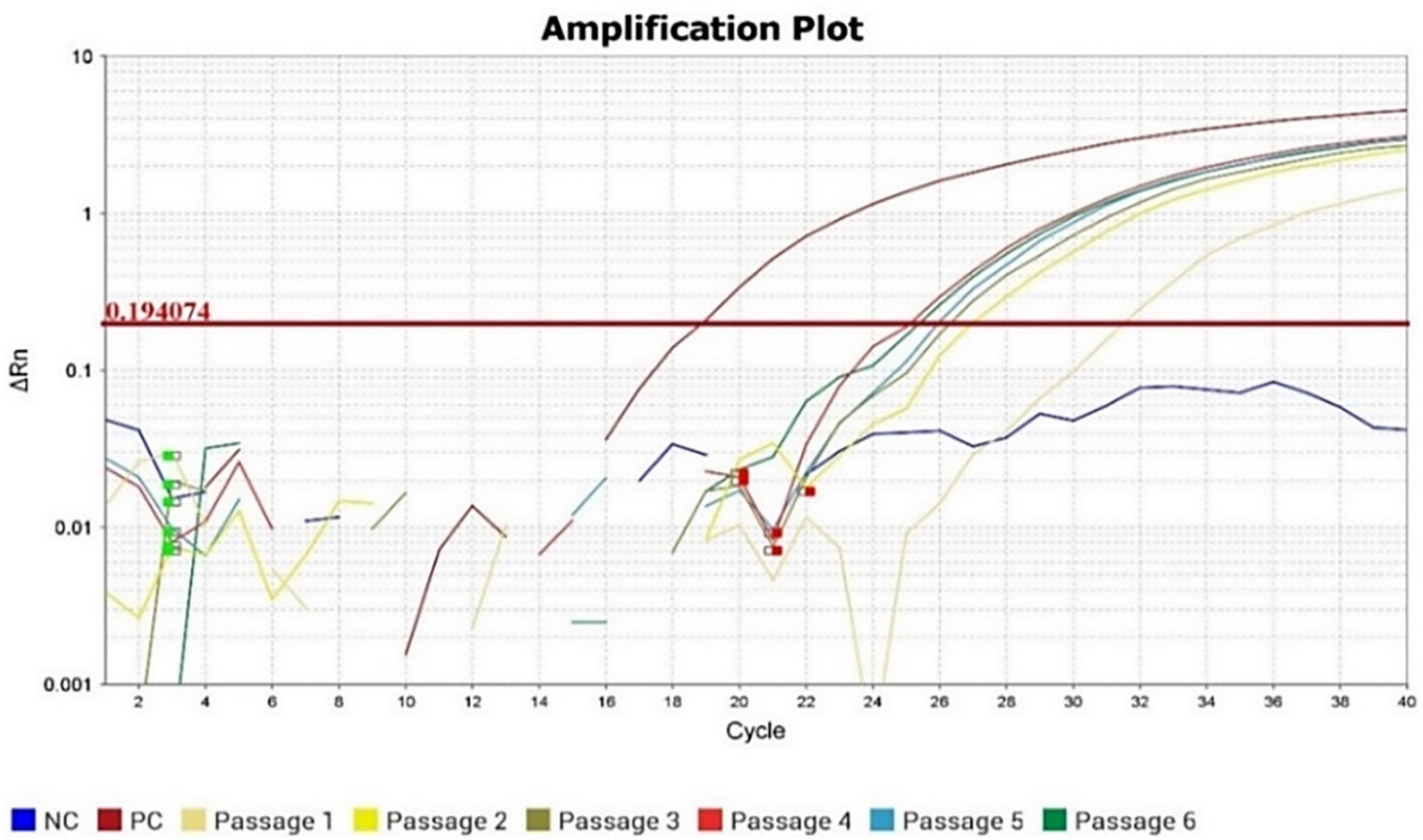
RT-qPCR amplification plot for the RNA from JEV passages 1 to 6. (NC – Negative Control, PC – Positive Control).

**Figure 4:**
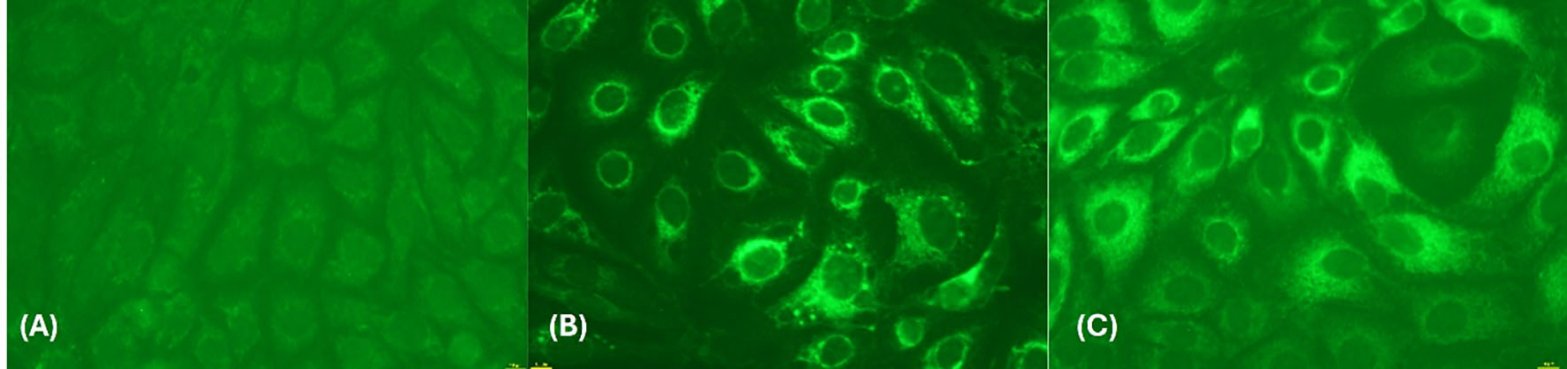
Immunofluorescence assay for JEV detection. **(A)** Mock infected PS cells (40x), **(B)** Positive control (JEV SA14-14-2) (40x) **(C)** JEV isolated from nasal swab of pig no. 4 (40x) at passage 5.

Confirmation of viral presence in all the six cell culture passages of the isolated virus was done by RT-qPCR, with progressive reduction in Ct values across successive passages (**Figure 3**). The *5′UTR–prM* gene (passage 4) and complete envelope gene (passage 6) was sequenced, and the presence of JEV antigen was further confirmed by IFA in passage 5 (**Figure 4**). The nucleotide sequences were deposited to GenBank with the accession numbers PX489696 (5′ *UTR–prM* region) and PX489697 (full-length envelope gene).

### 3.3 Phylogenetic analysis

**Figure 5:**
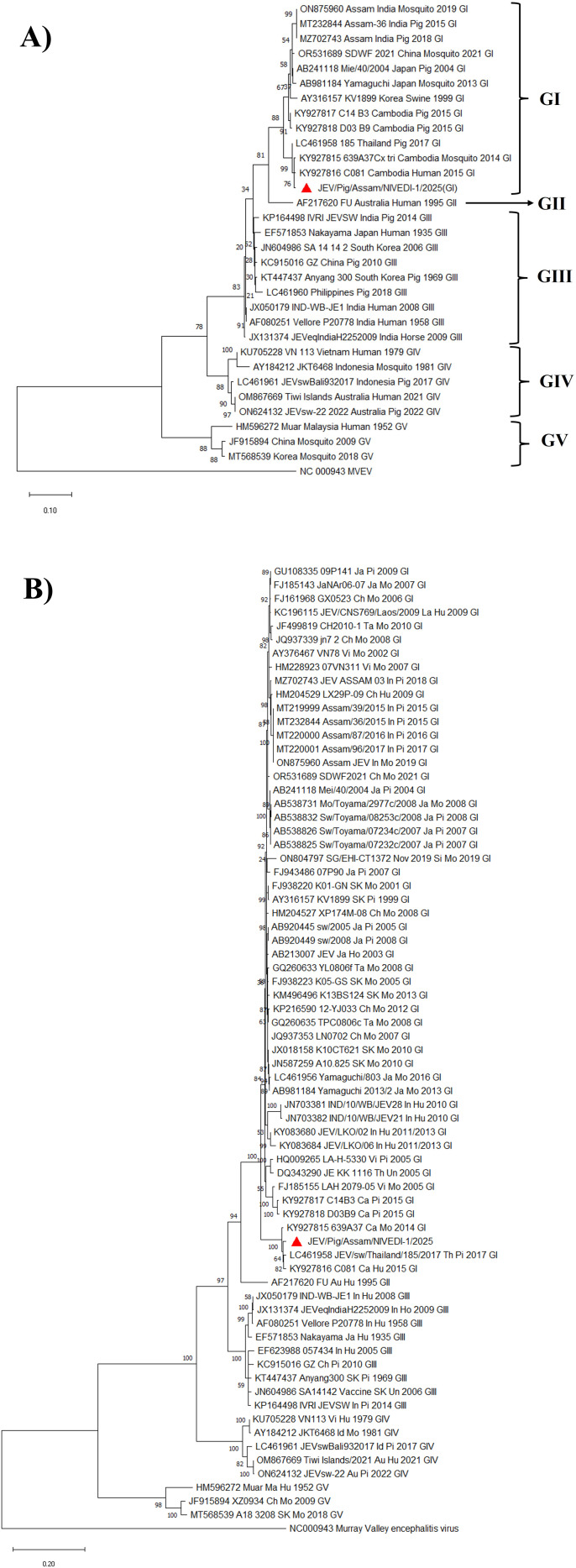
Phylogenetic tree of JEV isolate based on **(A)** 5’*UTR-prM* region nucleotide sequence, **(B)** Based on full length envelope gene nucleotide sequence. The JEV strains are labelled using accession number–strain name–geographical origin-host-year-genotype. The Host details are mentioned as Hu-Human, Ho-Horse, Pi-Pig, Mo-Mosquito, Un-Unknown. The location names are mentioned as In-India, Ja-Japan, SK-South Korea, Ch-China, Vi-Vietnam, Ta-Taiwan, Th-Thailand, Ca-Cambodia, Au-Australia, Id-Indonesia, Ma-Malaysia. Genotypes are mentioned as GI-GV. The scale bar indicates the nucleotide substitution per site. Red triangle indicates JEV sequence from the current study.

The PCR amplicon of the *5′UTR–prM* (505 bp) and envelope gene (1500 bp) obtained from passage 4 and 6, respectively were sequenced in triplicate, and consensus nucleotide sequence for each gene was derived. Phylogenetic comparison with representative JEV sequences of GI–GV retrieved from GenBank revealed that the isolate belonged to GI. Based on the *5′UTR–prM* region the isolate formed a distinct cluster with GI strains reported from Thailand and Cambodia, showing 99% nucleotide identity (**Figure 5**, **Table 3**). Comparatively, it shared 95% nucleotide identity with previously reported JEV isolates from Assam, India (**Table 3**). Similarly, based on envelope gene based phylogenetic analysis, the isolate clustered with GI isolates from Thailand and Cambodia (**Figure 5**).

**Table 3:** Percent identify of JEV 5’*UTR-prM* region nucleotide sequences from different geographical origin and host with the JEV isolated in the present study.

Accession	Genotype	Year	Host	Geographical location	Percent identity	Mismatches
LC461958.1	GI	2017	Pig	Thailand	99.21	4
KY927815.1	GI	2014	Mosquito	Cambodia	99.21	4
KY927816.1	GI	2015	Human	Cambodia	99.21	4
KY927818.1	GI	2015	Pig	Cambodia	96.04	20
KY927817.1	GI	2015	Pig	Cambodia	95.84	21
OR531689.1	GI	2012	Mosquito	China	95.45	23
AB241118.1	GI	2004	Pig	Japan	95.25	24
AB981184.1	GI	2013	Mosquito	Japan	95.05	25
ON875960.1	GI	2019	Mosquito	Assam, India	95.05	25
MT232844.1	GI	2015	Pig	Assam, India	95.05	25
MZ702743.1	GI	2018	Pig	Assam, India	95.05	25
AY316157.1	GI	1999	Pig	Korea	94.85	25
KP164498.2	GIII	2014	Pig	UP, India	91.29	43
AF217620.1	GII	1995	Human	Australia	91.18	43
JX131374.1	GIII	2009	Horse	Haryana, India	90.91	43
ON624132.1	GIV	2022	Pig	Australia	87.72	57
HM596272.1	GV	1952	Human	Malaysia	87.66	47
LC461961.1	GIV	2017	Pig	Indonesia	87.13	60
MT568539.1	GV	2018	Mosquito	Korea	82.7	80

The nucleotide and amino acid sequence identity analysis of the envelope gene revealed that the JEV GI isolated in the current study shared 90-92% and 96-98% identity at the nucleotide and amino acid level, respectively with the previous JEV GI sequences from India (**Table 4**). Further, the nucleotide mutations in envelope gene associated with attenuation of virulence was evaluated by comparing the amino acids of the isolated JEV strain with that of in the JEV SA 14 14 2. It was found that, the JEV GI isolate possessed all the eight critical amino acids found in the neurovirulent JEV strains (**Table 5**). To the best of our knowledge, this represents the first isolation of a JEV GI strain from the nasal swab of a naturally infected pig in India.

**Table 4:** Nucleotide and amino acid similarity matrix of the envelope gene of the JEV isolated in the present study (NIVEDI-1/2025 (GI) with the other JEV GI strains from India.

The GenBank accession numbers of JEV envelope gene sequences from India	NIVEDI-1/2025 (GI)	MT219999	MT220000	MT220001	MT232844	MZ702743	JN703381	JN703382	KY083680	KY083684	ON875960
NIVEDI-1/2025 (GI) Pig		0.98	0.98	0.98	0.98	0.976	0.972	0.964	0.982	0.976	0.98
MT219999 Pig	0.92		1	1	1	0.996	0.988	0.98	0.994	0.988	1
MT220000 Pig	0.92	0.998		1	1	0.996	0.988	0.98	0.994	0.988	1
MT220001 Pig	0.921	0.999	0.999		1	0.996	0.988	0.98	0.994	0.988	1
MT232844 Pig	0.92	0.997	0.997	0.998		0.996	0.988	0.98	0.994	0.988	1
MZ702743 Pig	0.926	0.989	0.989	0.99	0.989		0.984	0.976	0.99	0.984	0.996
JN703381 Human	0.904	0.94	0.94	0.941	0.941	0.947		0.992	0.99	0.984	0.988
JN703382 Human	0.9	0.938	0.938	0.939	0.939	0.945	0.993		0.982	0.976	0.98
KY083680 Human	0.922	0.964	0.964	0.965	0.965	0.97	0.957	0.955		0.994	0.994
KY083684 Human	0.917	0.952	0.952	0.952	0.952	0.958	0.942	0.94	0.984		0.988
ON875960 Mosquito	0.921	0.999	0.999	1	0.998	0.99	0.941	0.939	0.965	0.952	

The nucleotide similarities are shown above the diagonal and the amino acid identities are below the diagonal.

**Table 5:** Amino acids associated with neurovirulence property of isolated JEV in comparison to the vaccine strain (Amino acids are numbered from the first amino acid of the envelope protein).

JEV strain	Amino acid positions in the envelope protein							
	107	138	176	177	264	279	315	439
JEV SA 14 14 2	F	K	V	A	H	M	V	R
JEV/Pig/Assam/NIVEDI-1/2025 (GI)	L	E	I	T	Q	K	A	K

### 3.4 *In-vitro* growth kinetics

**Figure 6:**
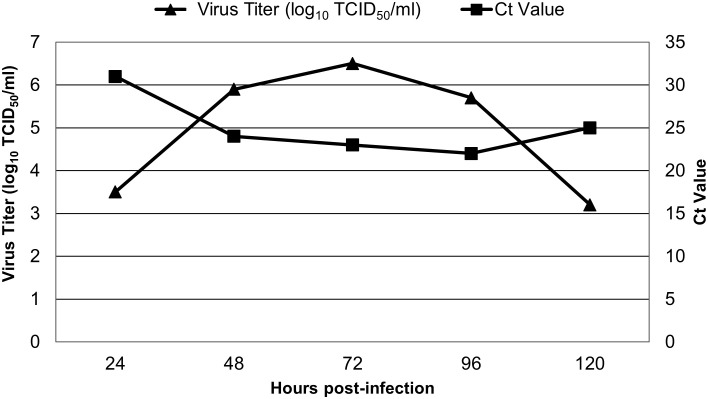
The plaques produced by laboratory maintained JEV GIII (H225) isolate and the JEV GI isolated in the present study.

The replication kinetics of JEV/Pig/Assam/NIVEDI-1/2025 (GI) in PS cells demonstrated a progressive increase in virus titer over time. The viral titer was 10^4.45^ TCID_50_/mL at 24 h post infection (p.i.), reached a peak of 10^6.5^ TCID_50_/mL at 72 h p.i., and declined thereafter to 10^3.25^ TCID_50_/mL at 120 h p.i. RT-qPCR analysis of RNA extracted from cell lysates and culture supernatants at sequential time points (24–120 h p.i.) showed decreasing Ct values from 31 at 24 h to 22 at 96 h, with a slight increase to 25.4 at 120 h (**Figure 6**). The highest viral titer (10^6.5^ TCID_50_/mL) corresponded with the Ct value of 23 at 72 h p.i., indicating peak replication during mid-logarithmic growth phase.

### 3.5 Phenotypic characterization of viral plaques

**Figure 7:**
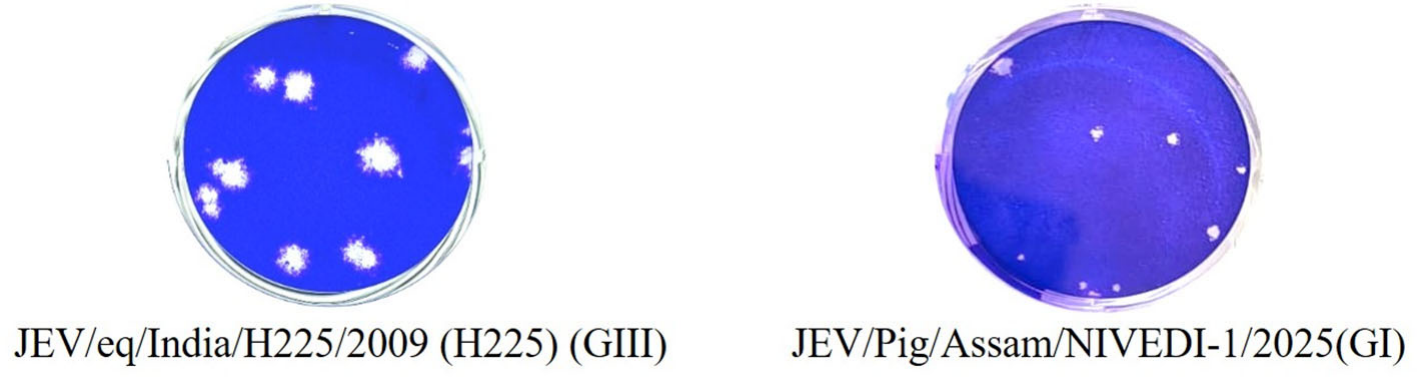
Replication kinetics at different time points after infection in PS cell line using RT-qPCR and virus titration of JEV GI isolate at passage 6.

The JEV/Pig/Assam/NIVEDI-1/2025 (GI) isolate produced smaller and more variable plaques compared with the GIII reference strain JEV/eq/India/H225/2009 (H225) maintained in our laboratory. The mean plaque diameter for the reference strain was 2.68 ± 0.48 mm, whereas the GI isolate produced plaques averaging 1.88 ± 0.56 mm (**Figure 7**). The difference in mean plaque size between the two genotypes was statistically significant (unpaired t-test, p < 0.01), suggesting phenotypic variation between JEV genotypes I and III.

### 3.6 Serological screening

The four pig serum samples from Farm A collected on 29 May 2025 were negative for neutralizing antibodies to JEV (MNT titers <1:10) (**Table 1**). Of the 30 serum samples screened during June 2025 from the same village, 11 (37%) were positive for JEV IgG.

## 4 Discussion

In the present study, we report the first isolation and characterization of a JEV GI from the nasal swab of a naturally infected pig in Assam, India. Previous studies in India have reported isolation of genotype III (GIII) JEV strains from clinical specimens such as blood, serum, and tissues of pigs and horses (Geevarghese et al., 1987; Gulati et al., 2012; Desingu et al., 2016). However, to the best of our knowledge, there are no prior reports of successful JEV GI isolation from nasal swab of pig, marking this as a novel finding.

Our results corroborate earlier experimental studies demonstrating that infectious JEV can be shed through the oro-nasal route in pigs for approximately 4–6 days post-infection, depending on the inoculation route (Ricklin et al., 2016; Redant et al., 2020). The present finding of virus isolation from a nasal swab of a viremic pig reinforces these observations under natural conditions. Ricklin et al., 2016 also demonstrated that naïve pigs co-housed with infected animals became infected via oro-nasal transmission, and viral RNA remained detectable in nasal swabs beyond the viremic phase. More recent studies further confirm that oro-nasal fluid and nasal swabs collected from both experimentally and naturally infected pigs are reliable diagnostic samples for detecting JEV RNA (Ricklin et al., 2016; Lyons et al., 2018; Chiou et al., 2021). Further, Chiou et al., 2021 showed that oro-nasal fluid could be more sensitive than blood or PBMCs for JEV detection.

The detection and isolation of JEV from nasal swab have a broader epidemiological implication, as JEV could be transmitted from pig-to-pig through oronasal route without the involvement of mosquitoes (Ricklin et al., 2016). It means, JEV could transmit between pigs in the season and geographical locations which are unfavorable to mosquitoes and JEV can be maintained in pig herds without the requirement of vectors (Ricklin et al., 2016). Hence, to prevent the persistence of the JEV in the herd through the sustained oro-nasal transmission cycle, all-in-all-out system of pig rearing could be an alternative (Chiou et al., 2021). However, in this study, since only one pig was positive for JEV detection and isolation in nasal swab specimen from only one herd, further field studies in large scale are warranted to evaluate the nasal shedding of JEV in naturally infected pigs.

The phylogenetic analysis of the *5′UTR–prM* region and complete envelope gene revealed that the JEV isolate clustered with GI strains from Thailand and Cambodia confirming the identity of the isolate as GI. Further, there was 90-92% and 96-98% identity at the nucleotide and amino acid level in the envelope gene with the previous GI isolate from India. This confirms genotype diversity and possible evolution of JEV. Although, the JEV strains have been classified into five genotypes based on Envelope gene nucleotide sequence, it has also been established that nucleotide sequences of the prM and capsid–pre-membrane regions (ranging from 227–346 nt) also provide sufficient variability for JEV genotype classification (Chen et al., 1992; Ali and Igarashi, 1997; Sapkal et al., 2007; Mote et al., 2023). In our study both the 5′*UTR–prM* region and complete envelope gene base phylogenetic analysis placed the JEV isolate into the GI cluster re-affirming the utility of both the genes in genotypic classification of JEV.

Our study detected JEV GI from a Pig in Assam. The JEV GIII was historically the predominant genotype circulating in pigs in India, including Assam (Pegu et al., 2019; Mote et al., 2023) barring detection of GI in few studies (Datey et al., 2020; Raut et al., 2021). However, our study provides the first virological evidence through virus isolation supporting the co-circulation of JEV GI along with GIII in pigs in Assam. Similarly, JEV GI detections in humans have been reported in Uttar Pradesh (2009) and West Bengal (2010) confirming the co-circulation of GI alongside GIII in these states (Fulmali et al., 2011; Sarkari et al., 2012). The genotype shift from GIII to GI has been observed across several Asian countries. For example, in South Korea and Japan, GI largely replaced GIII during the early 1990s (Yun et al., 2010), while in China, both genotypes continue to co-circulate in swine herds (Chai et al., 2018). The genotype shift from GIII to GI and the re-emergence of GV after several decades has been a cause of concern since all the existing human and animal JE vaccines are based on GIII strains and there is 8% to 11% amino acid difference among GV, GI and GIII strains (Tajima et al., 2015). Although, the existing GIII based vaccines have shown to provide cross protection, variations in neutralizing antibody titers and duration of immunity have been reported (Erra et al., 2013). Experimental data indicate that the human JE vaccines elicit lower neutralizing antibody titers against GV (seroconversion rate 35%) compared with GI (96%) and GIII (100%) (Cao et al., 2016). Therefore, active virological surveillance in pigs is essential to track the genotype shifts and provide evidence-based inputs for vaccination strategy and developing broadly protective vaccines against heterologous JEV genotypes. The deduced amino acid sequence of the envelope gene identified that the JEV GI strain possessed eight critical amino acids (Positions, 107, 138, 176, 177, 264, 279, 315, 439), the major determinant of JEV neuro-virulence. Though no pathogenicity study in animal models was done in the present study, the presence of the exact eight amino acids in this isolate confirmed that there was no mutation leading to the virus attenuation.

Given the paucity of data on JEV GI isolates from India, we further characterized the isolate’s replication kinetics and phenotypic traits *in vitro*. The GI isolate produced smaller, variable plaques compared with the GIII reference strain (p < 0.01) and reached a peak titer of 10^6.5^ TCID_50_/mL at 72 h post-infection, which corresponded to an RT-qPCR Ct value of 23. These results are consistent with previous studies where GI strains demonstrated high replication efficiency in porcine, avian, and mosquito cell lines compared with human-origin cells. The greater adaptability of GI strains to multiple host cell types may explain the increased number of GI isolations from pigs and mosquitoes relative to humans (Han et al., 2014; Do et al., 2016; Xiao et al., 2018).

With limited sample size of 30 pigs, the sero-positivity recorded in this study was 37%. Previous study had identified sero-positivity of 11.5% in pigs from the same district (Baruah et al., 2018). In Assam, the human JE cases reach peak in the month of July and the detection of JEV in pigs at the end of May month in this study correlate well with the observation that, the human JE cases appear three to four weeks after the JEV detection in pigs (Borah et al., 2013; Handique et al., 2014; Kumar et al., 2025). Both the pigs’ farms screened in the present study have stagnant water bodies next to them. Upon interaction with the owner of the pig farms, it was found that wild bird activity was present around the farm. The presence of water bodies, rice paddies around the pig farms and wild bird exposure have been identified as an important risk factor for JEV infection for pigs in Assam (Hussain et al., 2022; Rajkhowa et al., 2022).

In conclusion, our study provides the virological evidence that JEV GI is circulating in Assam and there is need for strengthened JEV surveillance in swine to monitor genotype shifts, understand viral evolution, and generate field isolates critical for vaccine evaluation and preparedness against emerging JEV genotypes. The study also demonstrates the feasibility of using nasal swabs for virus detection and isolation thereby providing evidence for the presence of JEV in nasal secretion of naturally infected pigs.

## Data Availability

The datasets presented in this study can be found in online repositories. The names of the repository/repositories and accession number(s) can be found below: https://www.ncbi.nlm.nih.gov/genbank/, PX489696 https://www.ncbi.nlm.nih.gov/genbank/, PX489697.
